# Characterization of Fecal Microbiota and Serum Metabolome Variations Across Different Gestational Stages in Hu Sheep

**DOI:** 10.3390/ani16142149

**Published:** 2026-07-11

**Authors:** Lanlan Li, Huibin Shi, Saiqiao Wang, Xueru Dou, Pengcheng Wang, Heng Fan, Xingchen Liu, Xiaoyin Zhang, Kai Quan, Yuqin Wang

**Affiliations:** 1 College of Animal Science and Technology, Henan University of Science and Technology, Luoyang 471023, China; lanlanli112394@163.com (L.L.); wangsaiqiao2639@163.com (S.W.); 13343795208@163.com (X.D.); wang13693870652@163.com (P.W.); fh2622652889@163.com (H.F.); 13333893130@163.com (X.L.); zhangxiaoyin0426@163.com (X.Z.); 2 Henan Key Laboratory of Livestock and Poultry Genetic Improvement and Healthy Breeding, College of Animal Science and Technology, Henan University of Animal Husbandry and Economy, Zhengzhou 450046, China; huibinshi0715@163.com

**Keywords:** Hu sheep, pregnancy stages, gut microbiota, serum metabolomics, multi-omics integration, metabolic adaptation

## Abstract

This study investigated the variations in the fecal microbiota and serum metabolome of prolific Hu sheep across different gestational stages to understand their physiological relationships. Fecal and blood samples were collected from 24 multiparous ewes across four stages: non-pregnant and gestational days 55, 85, and 110. Fecal microbial communities were analyzed via 16S rRNA gene sequencing, and serum metabolic profiles were assessed using liquid chromatography–tandem mass spectrometry (LC-MS)-based untargeted metabolomics. Serum biochemical analysis showed that pregnant ewes had decreased urea concentrations (*p* < 0.05), an early-gestation peak in total cholesterol, and a mid-gestation peak in triglycerides (*p* < 0.05). Fecal microbiota analysis indicated higher alpha diversity during gestation than in the non-pregnant stage (*p* < 0.05), with *Firmicutes* as the dominant phylum and stage-specific variations in genera such as *Negativibacillus* and *Monoglobus*. Metabolomics analysis identified 68 differential metabolites primarily assigned to lipid, amino acid, and steroid hormone pathways. Procrustes and Spearman correlation analyses showed statistical concordance between the fecal microbial community structure and the serum metabolome. Specific genera, including *UCG-005*, *Alistipes*, and unclassified *Lachnospiraceae*, correlated positively with metabolites such as pregnanediol 3-O-glucuronide and specific sphingomyelins. In conclusion, the progression of pregnancy in Hu sheep is characterized by concurrent shifts in the fecal microbiota and serum metabolites. These concurrent variations correlate with host nitrogen reallocation and lipid parameters, providing baseline reference data for the nutritional management of gestating ewes. These results provide a useful reference for future studies investigating maternal physiology, nutrition, and microbiome dynamics in prolific sheep breeds.

## Simple Summary

Gestation requires significant nutrient redistribution in female animals, but the relationships between intestinal microbes and blood metabolic profiles during different pregnancy stages are not fully understood in sheep production. This study evaluated how the fecal microbial communities and blood biochemical parameters vary concurrently across different periods of pregnancy in Hu sheep. The results showed that pregnant ewes exhibit increased fecal microbial diversity and shifts in blood indicators, including decreased urea concentrations and stage-specific variations in cholesterol and triglycerides. Statistical analyses identified distinct correlations between specific gut bacteria and circulating metabolites related to lipids, amino acids, and hormone derivatives. These findings provide baseline data regarding the physiological and microbial variations in gestating ewes. This information is valuable to the livestock industry as it offers empirical reference points that can be considered when designing stage-specific diets and monitoring the physiological status of pregnant ewes under intensive management conditions.

## 1. Introduction

Hu sheep are an indigenous breed in China characterized by high prolificacy (frequent twin or triplet litters), early sexual maturity, and excellent adaptability to intensive stall-feeding systems, serving as a primary maternal line genetic resource for the modern mutton meat industry [1]. Under intensive management, the nutritional status of ewes during gestation influences fetal development and the overall economic efficiency of sheep operations [2]. Gestation introduces physiological and metabolic changes to the maternal body, including shifts in endocrine hormones, immune parameters, and nutrient partitioning to support fetal growth [3]. Metabolic perturbations during this period can lead to adverse pregnancy outcomes, such as abortion or maternal mortality [4]. Characterizing these stage-specific physiological and metabolic variations is essential to replace traditional static feeding protocols with dynamic phase-feeding regimens, thereby optimizing reproductive efficiency and preventing nutritional metabolic disorders.

The mammalian gastrointestinal tract acts as a site for nutrient digestion and hosts a microbial ecosystem. These microbial communities participate in host energy metabolism, immune system development, and pathogen resistance [5]. In ruminants like sheep, the gastrointestinal microbiota participates in the degradation of complex plant fibers [6,7]. The gut microbiome changes in response to host physiological states, such as age, diet, and stress, and the fecal microbiota reflects the microbial status of the lower intestinal environment [8]. During pregnancy, gut microbes ferment dietary components to produce short-chain fatty acids (SCFAs), which supply energy to the mother and may influence fetal development via immunomodulatory or other physiological pathways [9,10,11]. However, studies evaluating the stage-specific changes in the gut microbiome throughout the entire gestational process in Hu sheep remain limited.

Serum metabolomics allows for the profiling of endogenous small-molecule metabolites, reflecting the physiological phenotype resulting from environmental and internal stimuli [12,13]. Liquid chromatography–tandem mass spectrometry (LC-MS)-based untargeted metabolomics has been applied in ruminants to evaluate nutritional interventions, monitor health status, and assess reproductive functions [14]. In pregnant animals, variations in the serum metabolic profile, particularly lipid and glucose pathways, assist in monitoring maternal–fetal health and identifying potential complications [15,16,17]. Microbial metabolites can cross the intestinal barrier into the host circulation, interacting with host metabolic pathways [18]. Multi-omics integration profiling of these networks during pregnancy in prolific Hu sheep has been limited.

Based on this background, key biological hypotheses were tested regarding whether stage-specific changes in the maternal gut microbiota correlate with variations in the host serum metabolome and systemic physiological parameters. To evaluate these associations, this study used prolific Hu sheep managed under intensive conditions. Fecal and blood samples were collected across four stages: non-pregnant and gestational days 55, 85, and 110. By combining serum biochemical assays, 16S rRNA gene sequencing [5], and LC-MS-based untargeted metabolomics [3], this study aimed to: (1) describe the trajectories of the serum metabolic profile across gestational stages; (2) assess the variations in the gut microbial community composition; and (3) evaluate the statistical correlations between specific microbial taxa and host metabolites, such as hormones, lipids, and amino acids. These findings are intended to provide empirical reference data regarding the metabolic and microecological shifts in gestating Hu sheep. Practically, this baseline information offers field-level guidance for farm managers and feed manufacturers to refine total mixed ration (TMR) formulations, enabling precision nutrient adjustments that align with the distinct physiological requirements of different pregnancy phases.

## 2. Materials and Methods

### 2.1. Experimental Animals and Experimental Design

This study was approved by the Institutional Animal Care and Use Committee of Henan University of Science and Technology (Approval No. HAUSTEAW-2021-C00227) and conducted at the Henan Kunyuan Agriculture and Animal Husbandry Hu Sheep Breeding Farm (Qixianzhuang, Ruzhou, China). Twenty-four healthy, multiparous Hu ewes (3 to 4 years of age) with similar initial body weights (50.6 ± 2.3 kg; *p* > 0.05) were randomly assigned to four experimental groups (*n* = 6 per group) representing distinct physiological stages: non-pregnant control (Group A), day 55 of gestation (Group B), day 85 of gestation (Group C), and day 110 of gestation (Group D). The animal trial was conducted concurrently between March 2023 and September 2023 to minimize seasonal and environmental confounding factors. Estrus synchronization followed by artificial insemination (AI) was performed on all ewes to precisely determine and record the exact day of conception and gestational stages. Ewes were housed in pens under uniform management and fed the exact same total mixed ration (TMR) *ad libitum* throughout the experimental period. The diet was formulated according to the national feeding standards for meat-producing sheep (NY/T 816-2021) [19].

### 2.2. Sample Collection

Before morning feeding on the designated sampling days, 5 mL of blood was collected from the jugular vein of each ewe using vacuum tubes containing a coagulant. Blood samples were allowed to clot at room temperature for 30 min, centrifuged at 2500 rpm for 10 min at 4 °C, and the separated serum was aliquoted and stored at −80 °C for biochemical assays and metabolomics. Concurrently, fresh rectal fecal samples were collected via rectal palpation using sterile gloves, immediately transferred into cryovials, transported to the laboratory on dry ice, and stored at −80 °C for DNA extraction and sequencing.

### 2.3. Determination of Serum Biochemical, Antioxidant, and Immune Indices

Serum biochemical indices were determined using a Chemray 240 automated biochemistry analyzer (Rayto Life and Analytical Sciences Co., Ltd., Shenzhen, China). The specific commercial assay kits used, all manufactured by Rayto, included: total protein (TP, Cat. No. R03802), albumin (ALB, Cat. No. R03704), urea (UREA, Cat. No. R02902), uric acid (UA, Cat. No. R03102), triglycerides (TG, Cat. No. R02802), total cholesterol (T-CHO, Cat. No. R03004), low-density lipoprotein cholesterol (LDL-C, Cat. No. R03402), high-density lipoprotein cholesterol (HDL-C, Cat. No. R03504), alanine aminotransferase (ALT, Cat. No. R01502), aspartate aminotransferase (AST, Cat. No. R01702), alkaline phosphatase (ALP, Cat. No. R02102), and glucose (GLU, Cat. No. R02402).

Serum antioxidant indices, including total antioxidant capacity (T-AOC), superoxide dismutase (SOD), catalase (CAT), malondialdehyde (MDA), and glutathione peroxidase (GSH-PX), were measured using commercial assay kits (Beijing Bio-Tech Co., Ltd., Beijing, China) according to the manufacturer’s instructions. Serum immunoglobulins (IgA, IgG, and IgM) were quantified by enzyme-linked immunosorbent assay (ELISA) using commercial kits from Jiangsu Jingmei Biotechnology Co., Ltd. (Jiangsu, China).

### 2.4. Fecal 16S rRNA Gene Sequencing and Microbial Community Analysis

Total genomic DNA was extracted from fecal samples using the E.Z.N.A.^®^ Soil DNA Kit (Omega Bio-tek, Norcross, GA, USA). DNA concentration and purity were determined using a NanoDrop 2000 spectrophotometer (Thermo Scientific, Wilmington, DE, USA). The V3–V4 regions of the bacterial 16S rRNA gene were amplified by PCR using primers 338F (5′-ACTCCTACGGGAGGCAGCAG-3′) and 806R (5′-GGACTACHVGGGTWTCTAAT-3′). Purified PCR products were sequenced on the Illumina MiSeq PE300 platform (Shanghai Majorbio Bio-Pharm Technology Co., Ltd., Shanghai, China) using the NEXTFLEX Rapid DNA-Seq Kit. Raw reads were quality-filtered with fastp (Version 0.19.6) [20] and merged with FLASH (Version 1.2.11) [21]. Sequences were denoised using the DADA2 plugin [22] within QIIME2 (Version 2020.2) [23] to generate amplicon sequence variants (ASVs), and sequence depth was rarefied to 20,000 reads per sample. Taxonomic annotation was performed against the Silva database (v138) [24]. Alpha diversity indices were calculated using Mothur (Version 1.30.2) [25]. Beta diversity was evaluated by Principal Coordinate Analysis (PCoA) based on Bray–Curtis distances, with compositional variations tested by ANOSIM. Linear discriminant analysis Effect Size (LEfSe) identified differential bacterial taxa among groups (LDA score > 2.0, *p* < 0.05) [26]. PICRUSt2 (v2.2.0) [27] and Tax4Fun (Version 0.3.1) [28] predicted functional profiles based on the COG and KEGG databases, respectively.

### 2.5. Serum Untargeted Metabolomics Analysis

Thawed serum (100 μL) was mixed with 400 μL of pre-cooled methanol:water (4:1, *v*/*v*) containing 0.02 mg/mL internal standard, homogenized, and ultrasonicated for 30 min at 5 °C (40 kHz). Samples stood at −20 °C for 30 min and were centrifuged at 13,000× *g* for 15 min at 4 °C. Supernatants were analyzed using a UPLC-TripleTOF mass spectrometer (AB Sciex, Framingham, MA, USA) in positive (ESI+) and negative (ESI−) electrospray ionization modes. Quality control (QC) samples were prepared by pooling equal aliquots from all samples. Raw LC-MS data were processed using Progenesis QI (Version 2.3, Waters Corporation, Milford, MA, USA) for baseline filtering, peak identification, alignment, and correction. Retention was limited to variables with non-zero values in >80% of samples, followed by log_10_-transformed. Metabolites were annotated against the HMDB [29] and Metlin [30] databases. Principal Component Analysis (PCA) and Orthogonal Partial Least Squares Discriminant Analysis (OPLS-DA) were performed on the Majorbio Cloud Platform. Significant differential metabolites (DMs) were filtered based on a Variable Importance in Projection (VIP) > 1 in the OPLS-DA model and *p* < 0.05 in Student’s *t*-test. Pathway enrichment was analyzed using the KEGG database [31].

### 2.6. Multi-Omics Integration and Statistical Analysis

Serum biochemical, antioxidant, and immune indices were analyzed via SPSS 25.0 (SPSS Inc., Chicago, IL, USA). After data normality and homogeneity of variance verification, a One-way Analysis of Variance (ANOVA) evaluated the main effects of gestational stages, followed by Duncan’s multiple range test for post hoc comparisons. Data are expressed as Mean ± Standard Deviation (SD), with significance defined at *p* < 0.05 [32]. For multi-omics integration, Procrustes analysis evaluated the spatial concordance between the fecal microbial community structure and serum metabolite profiles based on PCA ordinations. Spearman’s rank correlation analysis calculated correlation coefficients between the differential microbial taxa and differential metabolites. Heatmaps were generated using R software (Version 4.5.2) [33] to present correlations between the microbial taxa and metabolic phenotypes.

## 3. Results

### 3.1. Dynamic Changes in Serum Biochemical Indices

Serum concentrations of aspartate aminotransferase (AST), alanine aminotransferase (ALT), albumin (ALB), alkaline phosphatase (ALP), glucose (GLU), total protein (TP), and globulin (GLB) did not show significant differences between the non-pregnant control (Group A) and the gestational stages (Groups B, C, and D) (*p* > 0.05; Table 1). However, significant variations were observed in nitrogen and lipid metabolism indicators across different stages. Serum urea (UREA) concentrations decreased with the progression of gestation, with the highest values observed in Group A and the lowest stable values found in Groups C and D (*p* < 0.05). For lipid parameters, total cholesterol (T-CHO), high-density lipoprotein cholesterol (HDL-C), and low-density lipoprotein cholesterol (LDL-C) peaked on day 55 of gestation (Group B) and were higher than those in Group A and later gestational stages (*p* < 0.05). Serum triglycerides (TG) were lowest in Group A and increased to their maximum concentration on day 85 of gestation (Group C; *p* < 0.05) before decreasing.

### 3.2. Serum Antioxidant and Immune Indices

Serum catalase (CAT) activity was highest in non-pregnant ewes (Group A) and decreased during gestation (Groups B, C, and D; *p* < 0.05; Table 2). Glutathione peroxidase (GSH-PX) activity increased on day 55 of gestation (Group B) and was higher than that on day 110 of gestation (Group D; *p* < 0.05). No significant differences were observed in SOD, SOD inhibition rate, MDA, and T-AOC among all experimental groups (*p* > 0.05). Serum concentrations of immunoglobulins IgA, IgG, and IgM remained stable, showing no significant differences between the non-pregnant and gestational stages (*p* > 0.05).

### 3.3. Characteristics and Differential Analysis of Serum Metabolic Profiles

**Figure 1:**
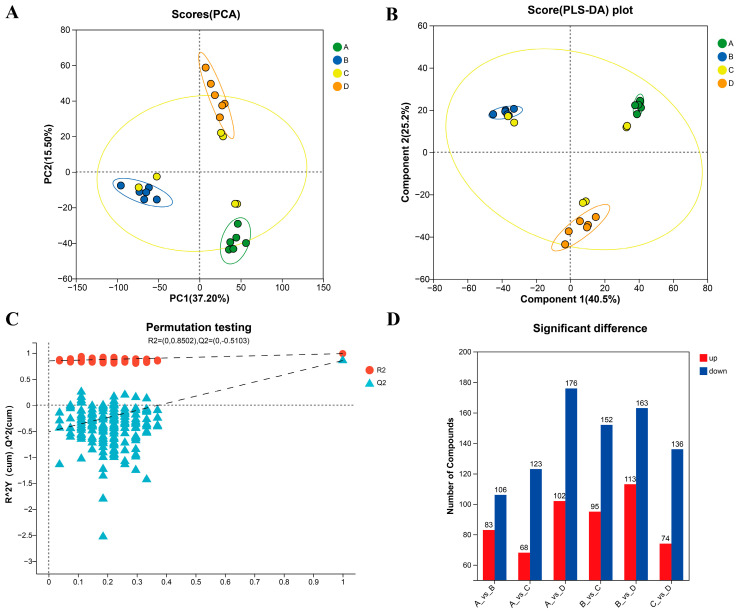
Multivariate statistical analysis and differential metabolite screening of the serum metabolome in Hu sheep across different gestational stages. (**A**) Unsupervised PCA score plot. (**B**) PLS-DA score plot. (**C**) Permutation test of the PLS-DA model with 200 iterations (R^2^ and Q^2^ intercepts are indicated on the plot). (**D**) Bar chart showing the number of significantly up-regulated (red) and down-regulated (blue) differential metabolites from pairwise comparisons.

Untargeted metabolomics analysis identified 2655 and 1999 metabolite features in positive and negative electrospray ionization modes, respectively. The unsupervised PCA score plot showed spatial separation among Groups A, B, and D, with Group C partially overlapping with the other groups (Figure 1A). The PLS-DA model showed spatial separation among all four experimental groups (Figure 1B), and permutation testing with 200 iterations yielded intercepts of *R*^2^ = 0.8502 and *Q*^2^ = −0.5103 (Figure 1C).

Based on a VIP > 1 and *p* < 0.05, 298 differential metabolites (DMs) were identified in positive ion mode and 258 DMs in negative ion mode (Table S1). Pairwise comparisons showed the number of altered metabolites between specific gestational stages (Figure 1D). Chemical classification showed that these DMs included lipids and lipid-like molecules (33.71%), organic acids and derivatives (20.83%), organoheterocyclic compounds (14.58%), benzenoids (8.52%), and organic oxygen compounds (8.14%; Figure S1A). KEGG pathway enrichment analysis indicated that the DMs were enriched in amino acid metabolism pathways (tryptophan metabolism, arginine biosynthesis, and valine, leucine, and isoleucine biosynthesis), energy metabolism (citrate cycle), and transmembrane transport (ABC transporters; Figure S1B).

### 3.4. Fecal Bacterial Microbiome

**Figure 2:**
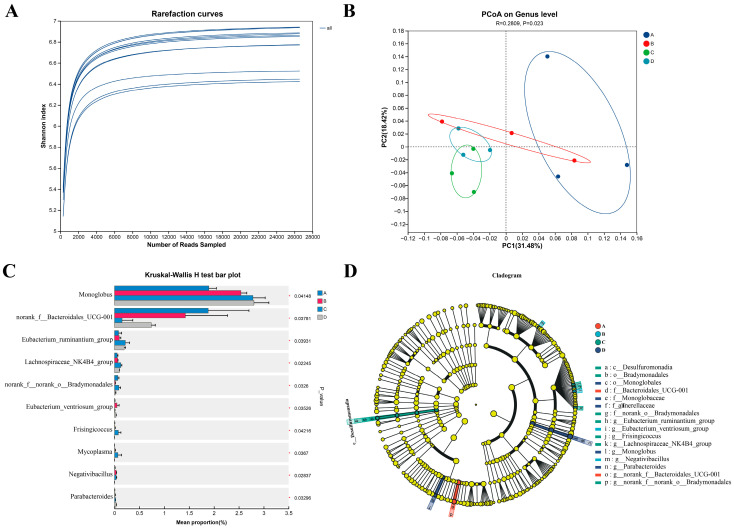
Alpha and beta diversity parameters and differential taxa analysis of fecal microbial communities in Hu sheep across different gestational stages. (**A**) Rarefaction curves based on the Shannon diversity index. (**B**) Principal coordinate analysis (PCoA) plot based on unweighted UniFrac distances at the genus level (R and *p* values derived from ANOSIM are indicated). (**C**) Bar plot of differential genera analyzed by the Kruskal–Wallis H test (*p* < 0.05). (**D**) Cladogram generated from LEfSe analysis showing the phylogenetic distribution of bacterial taxa with an LDA score > 2.0. Nodes with different colors represent taxa significantly enriched in the respective groups, while yellow nodes indicate taxa with no significant differences among groups.

High-throughput sequencing of the fecal 16S rRNA gene yielded 765,936 raw reads, with an average of 63,828 reads per sample (Table S2). Rarefaction curves based on the Shannon index reached plateaus (Figure 2A), and Good’s coverage values were above 99.00% for all samples (Table S3). For alpha diversity, all gestational groups (Groups B, C, and D) had higher community richness indices (Sobs, Ace, and Chao) and diversity index (Shannon) compared to the non-pregnant group (Group A) (*p* < 0.05), while the Simpson index was lower in the gestational groups (*p* < 0.05; Table S3). PCoA at the genus level showed that samples from Group A separated from those of Groups C and D, with minor overlap between Groups A and B (Figure 2B). ANOSIM confirmed variations in microbial composition across different stages (R = 0.2809, *p* = 0.023).

A total of 16 bacterial phyla were identified (Figure S2A), dominated by *Firmicutes* (62.01%) and *Bacteroidota* (29.67%). The relative abundance of *Firmicutes* was higher in the gestational groups (Groups B, C, and D) than in the non-pregnant group (*p* < 0.05), while no significant differences were found for *Bacteroidota*, *Spirochaetota*, *Proteobacteria*, *Verrucomicrobiota*, and *Patescibacteria* (*p* > 0.05; Table S4). At the genus level, 163 genera were detected, with the highest relative abundances found in *UCG-005* (8.34%), the *Christensenellaceae* R-7 group (7.81%), the *Rikenellaceae* RC9 gut group (7.64%), unclassified *Lachnospiraceae* (7.50%), and *norank_f__UCG-010* (5.93%; Figure S2B). Kruskal–Wallis H test (Figure 2C) and LEfSe analysis (Figure 2D) identified specific biomarker taxa across stages. The family *Bacteroidales* UCG-001 and its unclassified genus were enriched in Group A. The genus *Negativibacillus* and the *Eubacterium_ventriosum_group* were enriched in Group B. Seven taxa, including the class *Desulfuromonadia*, the order *Bradymonadales*, the *Eubacterium_ruminantium_group*, the genus *Frisingicoccus*, and the *Lachnospiraceae*_NK4B4_group, were enriched in Group C. The genus *Monoglobus*, the family *Tannerellaceae*, and the genus *Parabacteroides* were enriched in Group D.

**Figure 3:**
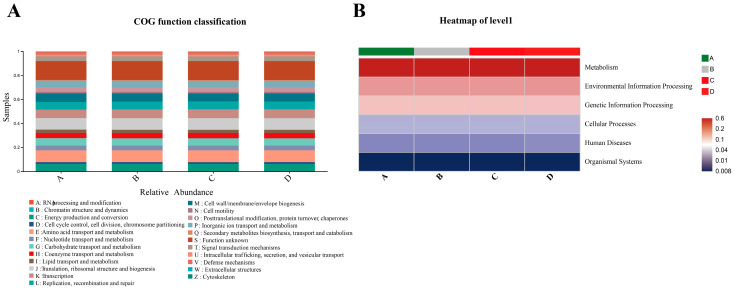
Predicted functional profiles of fecal microbial communities in Hu sheep across different gestational stages. (**A**) Stacked bar chart showing the relative abundance of Clusters of Orthologous Groups (COG) functional categories predicted by PICRUSt2. (**B**) Heatmap showing the relative abundance of KEGG Level 1 functional pathways predicted by Tax4Fun. The legend in (**A**) corresponds to specific COG functional modules. The color gradient in (**B**) represents the average relative abundance across samples, with red indicating higher abundance and blue indicating lower abundance.

Functional predictions via PICRUSt2 showed that the predicted genes were annotated in COG modules related to metabolism, information storage and processing, and cellular processes and signaling (Figure 3A). Tax4Fun functional pathways mapping indicated that the predicted genes were categorized into six KEGG Level 1 groups, with “Metabolism” maintaining the highest relative abundance across all stages (Figure 3B).

### 3.5. Multi-Omics Integration of the Gut Microbiome and Serum Metabolome

**Figure 4:**
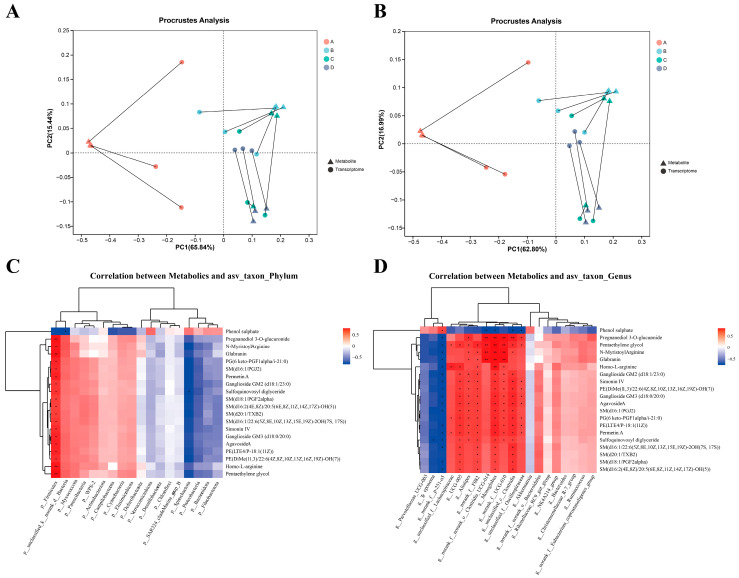
Correlation analysis between the fecal microbiota and differential serum metabolites in Hu sheep across different gestational stages. (**A**) Procrustes analysis at the phylum level based on PCA ordinations. (**B**) Procrustes analysis at the genus level based on PCA ordinations. In (**A**,**B**), triangles represent serum metabolites, circles represent microbial communities, and the length of the solid line connecting the two points indicates the residual magnitude. (**C**) Spearman correlation heatmap between the top 18 bacterial phyla and the top 20 differential metabolites. (**D**) Spearman correlation heatmap between the top 20 bacterial genera and the top 20 differential metabolites. Cells are colored based on correlation coefficients, with red indicating positive correlations and blue indicating negative correlations. Statistical significance thresholds are indicated by asterisks: * 0.01 < *p* < 0.05, ** *p* < 0.01.

Procrustes analysis showed correlation between the spatial ordinations of the fecal microbiota and serum metabolites at both the phylum (*p* = 0.014; Figure 4A) and genus (*p* = 0.018; Figure 4B) levels. At the phylum level, Spearman rank correlation analysis indicated that *Firmicutes* correlated positively (*p* < 0.05) with 19 metabolites, including sphingomyelins, gangliosides GM2/GM3, N-myristoyl arginine, homo-L-arginine, and pregnanediol 3-O-glucuronide (Figure 4C). *Spirochaetota* correlated negatively with sulfoquinovosyl diglyceride (*p* < 0.05), and an unclassified bacterial phylum (*norank_d__Bacteria*) correlated negatively with phenol sulphate (*p* < 0.05; Table S5).

At the genus level, *norank_f__p-251-o5* correlated negatively with 17 metabolites, including pregnanediol 3-O-glucuronide and pentaethylene glycol, and correlated positively with phenol sulphate (*p* < 0.05; Figure 4D). A group of nine genera, including unclassified *Lachnospiraceae*, *UCG-005*, *Akkermansia*, and *Alistipes*, correlated positively with these lipids, amino acid derivatives, and steroid hormone metabolites (Table S6).

## 4. Discussion

### 4.1. Gestational Adaptive Regulation of Serum Biochemical Indices

Serum biochemical indices reflect metabolic homeostasis, organ function, and nutritional status in livestock. Blood urea (UREA) is a product of protein catabolism, and its circulating concentration is inversely associated with the retention efficiency of dietary nitrogen. Previous studies indicate that variations in physiological states affect blood urea nitrogen levels [34,35,36]. In this study, maternal UREA concentrations decreased following conception and remained lower during mid-to-late gestation than in the non-pregnant stage. The reduction in serum urea concentrations may indicate alterations in maternal nitrogen metabolism during gestation, potentially reflecting changes in protein utilization and nutrient partitioning to support fetal development [37]. Furthermore, the concentrations of total protein (TP), albumin (ALB), and globulin (GLB) did not show significant fluctuations throughout the experimental period, indicating stable basal protein metabolism under the evaluated feeding conditions [38,39].

Maternal lipid and cholesterol levels are associated with progesterone synthesis and reproductive traits in ewes [40]. In this study, serum T-CHO, HDL-C, and LDL-C concentrations increased on day 55 of gestation (Group B) before decreasing in later stages. This early gestational hypercholesterolemia is concurrent with the initiation of placental vascular development and steroidogenesis [41]. Subsequently, maternal serum TG concentrations reached their highest values during mid-gestation (Group C), which represents a physiological phase of active lipid metabolism and maternal energy accumulation [42,43]. Therefore, monitoring these specific lipid profiles provides data for assessing maternal energy balance across different stages of pregnancy [4].

### 4.2. Gestational Adaptive Regulation of Maternal Antioxidant Defense and Immune Homeostasis

Gestation increases the basal metabolic rate in ewes, which alters the balance of reactive oxygen species (ROS) and the risk of oxidative stress. Circulating antioxidant biomarkers provide data regarding the physiological capacity to clear ROS and maintain cellular status. Superoxide dismutase (SOD) participates in dismutating superoxide radicals, while catalase (CAT) and glutathione peroxidase (GSH-Px) break down hydrogen peroxide and hydroperoxides [44,45,46]. In this study, serum CAT activity decreased during gestation compared to the non-pregnant stage, whereas GSH-Px activity showed a significant increase on day 55 of gestation (Group B) before declining. Serum MDA concentrations and T-AOC values remained stable across all groups. These findings suggest that individual antioxidant enzymes may respond differently during gestation, although the overall oxidative status appeared relatively stable.

Regarding immunity, serum immunoglobulins are involved in pathogen defense and systemic immune responses [47,48,49]. Immunoglobulins A (IgA), G (IgG), and M (IgM) are key components of humoral immunity. In this study, the circulating concentrations of these three major immunoglobulins did not show significant variations between the non-pregnant control and any of the gestational stages. The absence of significant changes in circulating immunoglobulin concentrations suggests that humoral immune status remained relatively stable throughout gestation.

### 4.3. Spatiotemporal Succession and Functional Associations of the Gut Microbiota

The gastrointestinal microbiota of ruminants participates in nutrient digestion, metabolic regulation, and barrier defense [50,51]. The structure of these microbial communities is modified by host factors such as gestational stage, diet, and age [52,53,54]. Evaluating these microbial variations assists in understanding their associations with host physiological shifts [55]. In agreement with previous findings identifying *Firmicutes* and *Bacteroidota* as the primary phyla in the ovine digestive tract [56], our results showed that the relative abundance of *Firmicutes* was higher during gestation than in the non-pregnant stage. Members of the phylum *Firmicutes* are involved in the fermentation of complex carbohydrates into short-chain fatty acids (SCFAs), and their higher abundance during pregnancy may reflect shifts in microbial functions associated with maternal metabolic adaptations.

LEfSe analysis identified specific microbial taxa associated with distinct stages of pregnancy. For example, the genus *Monoglobus*, which was enriched in late gestation (Group D), is involved in pectin degradation, potentially assisting the host in utilizing specific forage fibers. Changes in maternal microbial diversity may have implications for maternal–offspring microbial transmission [57]; however, this possibility was not evaluated in the present study. The observed increase in fecal microbial alpha diversity during pregnancy suggests variations in the microbial reservoir available during this physiological period. Functional predictions via PICRUSt2 and Tax4Fun indicated that these successional changes correlate with genes enriched in metabolism and environmental information processing pathways, consistent with the metabolic adjustments observed during gestation.

### 4.4. Associations Between the Fecal Microbiota and Serum Metabolome During Pregnancy

Metabolomics is applied in animal science to assess nutrient utilization [58], endocrine pathways [59], and metabolic responses to physiological or environmental shifts [60,61,62,63]. In ruminants, untargeted metabolomics has assisted in identifying metabolic variations associated with nutritional states or disorders [64,65,66]. In this study, untargeted metabolomics identified stage-specific variations in serum metabolites, particularly within lipid and amino acid derivative pathways, across different periods of pregnancy.

**Figure 5:**
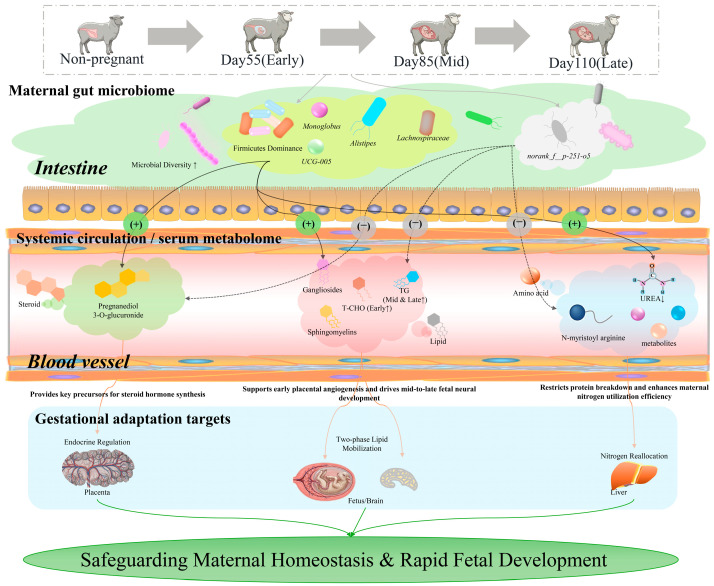
Working model summarizing the statistical correlations within the gut microbiota-serum metabolome axis during gestation in Hu sheep. The schematic diagram outlines the observed variations in the fecal microbiota and serum metabolites across four progressive stages (Non-pregnant, Day 55, Day 85, and Day 110 of gestation). In the top compartment, specific bacterial taxa are categorized based on their statistical profiles. Solid lines marked with (+) represent significant positive Spearman correlations between core hub genera (including *UCG-005*, *Alistipes*, and unclassified *Lachnospiraceae*) and major serum metabolite groups located in the middle circulation compartment. These correlated metabolites include steroid derivatives (e.g., pregnanediol 3-O-glucuronide), sphingomyelins, and amino acid derivatives (e.g., N-myristoyl arginine). Dashed lines marked with (−) represent negative statistical correlations associated with specific taxa, such as *norank_f__p-251-o5*. The arrows at the bottom indicate the alignment of these metabolite variations with measured host physiological indicators, specifically placental endocrine parameters, triglyceride and cholesterol repartitioning profiles, and decreased serum urea concentrations associated with hepatic nitrogen parameters. This model summarizes the structural concordance between lower-gut microbial succession and systemic metabolic indicators.

The composition of the gut microbiota is linked to the metabolic profile of the host, and variations in serum metabolites reflect these microbial activities. Using Procrustes and Spearman correlation analyses, this study evaluated the statistical relationships between fecal microbes and serum metabolites. The phylum *Firmicutes* and specific genera, including *UCG-005*, *Alistipes*, and unclassified *Lachnospiraceae*, had positive correlations with pregnanediol 3-O-glucuronide (a progesterone metabolite), certain sphingomyelins and gangliosides, and N-myristoyl arginine. These associations suggest that gestation-related changes in gut microbial composition occur alongside alterations in host metabolic profiles. Conversely, the genus *norank_f__p-251-o5* correlated negatively with several of these metabolites, suggesting a different relationship with host metabolic status. Collectively, these correlations indicate that the succession of the fecal microbiota is associated with systemic metabolic parameters during pregnancy. Based on these statistical linkages, we summarize these observed relationships in a working model (Figure 5).

Despite these findings, several limitations should be noted. First, the sample size (n = 6 per group) was relatively small, which warrants caution when interpreting the results. Second, fecal microbiota composition reflects the lower gastrointestinal tract and does not directly measure the pre-gastric fermentation dynamics occurring within the rumen. Third, this study was conducted at a single facility under a specific intensive management system; variations in diet composition or housing conditions may alter the observed microbial and metabolic trajectories. Future studies utilizing metagenomic sequencing and targeted metabolomics are needed to validate the specific predicted metabolic pathways identified here and to evaluate the effects of targeted dietary interventions on these microbial groups.

## 5. Conclusions

In summary, this study described the variations in the fecal microbiota and serum metabolites of Hu sheep across different gestational stages. The progression of pregnancy was characterized by a decrease in serum urea concentrations, a transient increase in cholesterol parameters during early gestation, and an increase in triglyceride concentrations during mid-gestation, alongside variations in serum catalase and glutathione peroxidase activities. Concurrently, the fecal microbiota showed higher alpha diversity indices during gestation than in the non-pregnant stage, with an observed enrichment of the genus *Monoglobus* in late gestation. Multi-omics integration via Procrustes and Spearman correlation analyses indicated statistical associations between specific lower-gut bacterial taxa (including *UCG-005*, *Alistipes*, and unclassified *Lachnospiraceae*) and circulating metabolites, such as pregnanediol 3-O-glucuronide, certain sphingomyelins, and amino acid derivatives.

These findings present the statistical correlations between changes in the fecal microbial community and shifts in the systemic metabolic profile during different periods of pregnancy. Additionally, specific stage-dependent indicators, such as serum phenol sulfate, were identified in relation to these physiological stages. These results provide a useful reference for future studies investigating maternal physiology, nutrition, and microbiome dynamics in prolific sheep breeds.

## Figures and Tables

**Table 1:** Serum biochemical indices of Hu sheep at different gestational stages.

Indices	Group A	Group B	Group C	Group D
AST (U/L)	60.53 ± 24.90	60.41 ± 9.30	68.64 ± 17.67	69.30 ± 11.11
ALT (U/L)	23.51 ± 7.57	23.39 ± 1.93	21.02 ± 2.86	18.86 ± 4.99
ALB (g/L)	24.17 ± 0.88	24.16 ± 1.99	21.90 ± 6.38	22.88 ± 7.29
ALP (U/L)	166.99 ± 43.72	156.01 ± 54.94	116.67 ± 17.63	138.84 ± 50.95
UREA (mmol/L)	3.01 ± 0.40 ^a^	2.30 ± 0.28 ^b^	0.39 ± 0.35 ^c^	0.35 ± 0.32 ^c^
T-CHO (mmol/L)	0.97 ± 0.45 ^b^	1.82 ± 0.39 ^a^	0.92 ± 0.40 ^c^	0.69 ± 0.44 ^c^
TG (mmol/L)	0.02 ± 0.00 ^b^	0.19 ± 0.05 ^ab^	0.38 ± 0.34 ^a^	0.28 ± 0.25 ^ab^
HDL-C (mmol/L)	0.55 ± 0.39 ^b^	0.82 ± 0.56 ^a^	0.34 ± 0.29 ^bc^	0.19 ± 0.25 ^c^
LDL-C (mmol/L)	0.11 ± 0.07 ^b^	0.33 ± 0.05 ^a^	0.15 ± 0.03 ^b^	0.13 ± 0.11 ^b^
GLU (mmol/L)	2.23 ± 1.48	2.49 ± 1.45	3.30 ± 1.06	3.37 ± 1.24
TP (g/L)	53.70 ± 7.11	52.52 ± 10.36	54.14 ± 6.91	51.47 ± 4.68
GLB (g/L)	30.46 ± 7.83	29.26 ± 10.20	32.51 ± 9.94	27.41 ± 7.08

Note: Data are expressed as mean ± standard deviation (SD). Means within a row with different superscript lowercase letters (a, b, c) differ significantly (*p* < 0.05), while means with the same or no superscript letters do not differ significantly (*p* > 0.05). Group A: non-pregnant ewes; Groups B, C, and D: pregnant ewes at days 55, 85, and 110 of gestation, respectively. Abbreviations: AST, aspartate aminotransferase; ALT, alanine aminotransferase; ALB, albumin; ALP, alkaline phosphatase; UREA, urea; T-CHO, total cholesterol; TG, triglycerides; HDL-C, high-density lipoprotein cholesterol; LDL-C, low-density lipoprotein cholesterol; GLU, glucose; TP, total protein; GLB, globulin.

**Table 2:** Serum antioxidant and immune indices of Hu sheep at different gestational stages.

Category	Indices	Group A	Group B	Group C	Group D
Antioxidant indices	CAT (U/mL)	47.99 ± 6.09 ^a^	26.08 ± 5.10 ^b^	19.42 ± 2.38 ^b^	28.12 ± 3.53 ^b^
	SOD (U/mL)	23.02 ± 0.21	22.49 ± 0.15	22.29 ± 0.45	22.80 ± 0.14
	SOD inhibition rate (%)	0.96 ± 0.01	0.94 ± 0.01	0.93 ± 0.02	0.95 ± 0.01
	GSH-PX (U/mL)	63.11 ± 23.64 ^ab^	98.77 ± 20.56 ^a^	44.15 ± 10.82 ^ab^	28.30 ± 7.55 ^b^
	MDA (nmol/mL)	36.88 ± 9.92	43.75 ± 5.64	43.13 ± 4.13	29.38 ± 5.34
	T-AOC (nmol/mL)	0.89 ± 0.02	0.90 ± 0.01	0.91 ± 0.01	0.90 ± 0.01
Immune indices	IgA (μg/mL)	19.94 ± 5.69	18.37 ± 3.38	20.59 ± 3.85	19.12 ± 1.89
	IgG (μg/mL)	943.74 ± 507.21	434.19 ± 137.29	827.62 ± 546.45	696.44 ± 266.89
	IgM (μg/mL)	9.48 ± 2.86	10.70 ± 2.46	9.35 ± 3.71	7.56 ± 1.16

Note: Data are expressed as mean ± standard deviation (SD). Means within a row with different superscript lowercase letters (a, b) differ significantly (*p* < 0.05), while means with the same or no superscript letters do not differ significantly (*p* > 0.05). Group A: non-pregnant ewes; Groups B, C, and D: pregnant ewes at days 55, 85, and 110 of gestation, respectively. Abbreviations: CAT, catalase; SOD, superoxide dismutase; GSH-PX, glutathione peroxidase; MDA, malondialdehyde; T-AOC, total antioxidant capacity; IgA, immunoglobulin A; IgG, immunoglobulin G; IgM, immunoglobulin M.

## Data Availability

The data are available from the corresponding author upon request.

## Supplementary Materials

The following supporting information can be downloaded at https://www.mdpi.com/article/10.3390/ani16142149/s1, Figure S1: Chemical classification and KEGG functional enrichment analysis of differential serum metabolites; Figure S2: Overall relative abundance composition of the intestinal microbial community in Hu sheep; Table S1: Number of detected metabolite features and differential metabolites in different pairwise comparisons under positive and negative ion modes; Table S2: Quality and basic statistics of 16S rRNA gene sequencing data of fecal samples in Hu sheep; Table S3: Alpha diversity indices of fecal microbiota in Hu sheep at different pregnancy stages; Table S4: Relative abundance (%) of dominant intestinal microbiota at the phylum level in Hu sheep at different pregnancy stages; Table S5: Spearman correlation coefficient matrix between dominant intestinal microbiota at the phylum level and differential serum metabolites.; Table S6: Spearman correlation coefficient matrix between top dominant intestinal microbiota at the genus level and differential serum metabolites.
