# Supplementary material for: Characterization of Fecal Microbiota and Serum Metabolome Variations Across Different Gestational Stages in Hu Sheep
Source: Animals (Basel). 2026 Jul 11;16(14):2149. doi: 10.3390/ani16142149 (PMC13406039; doi:10.3390/ani16142149)
Supplement: Supplementary file 1 [file animals-16-02149-s001.zip › Supplementary Figures.pdf]

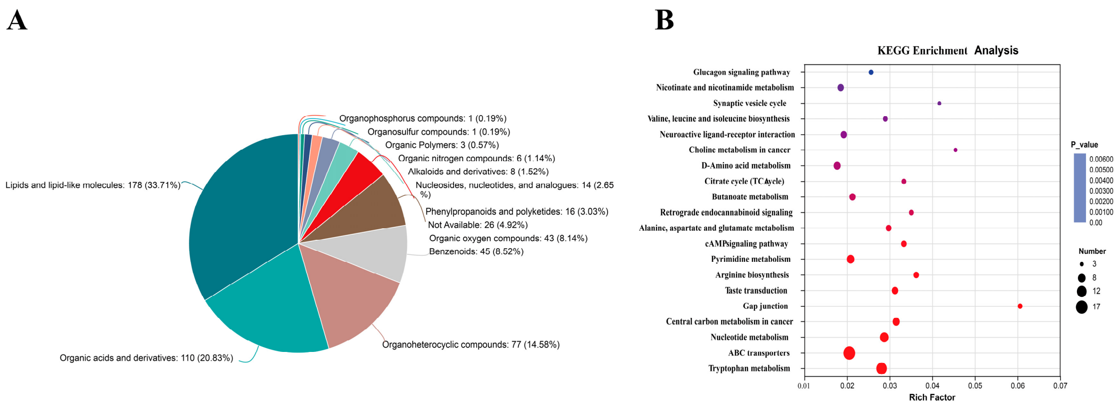

Figure S1. Chemical classification and KEGG functional enrichment analysis of differential serum metabolites. (A) Pie chart illustrating the chemical taxonomic classification and proportions of the identified differential metabolites, highlighting the predominance of lipids and lipid-like molecules (33.71%). (B) Bubble chart of KEGG pathway enrichment analysis for differential metabolites. The x-axis represents the rich factor, the size of the bubble corresponds to the number of enriched differential metabolites in the pathway, and the color gradient indicates the statistical significance (*p*-value).

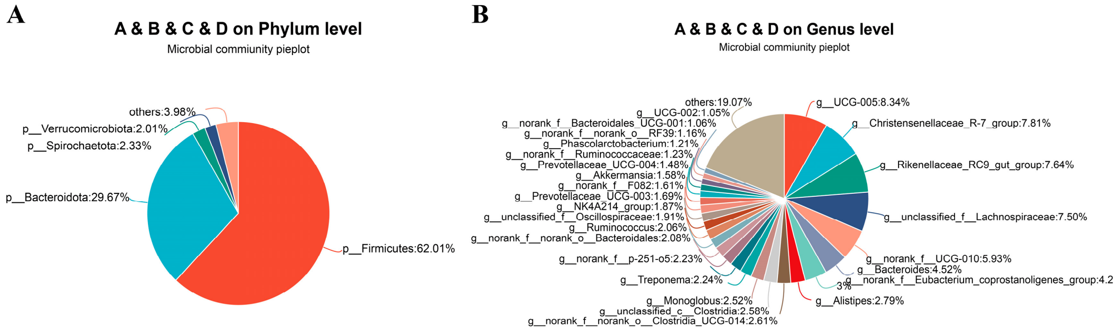

Figure S2. Overall relative abundance composition of the intestinal microbial community in Hu sheep. (A) Pie chart showing the relative abundance at the phylum level. (B) Pie chart showing the relative abundance at the genus level. Only the top dominant taxa are displayed, and the remaining low-abundance taxa are grouped into "others".
